# Patient safety in the operating room: an intervention study on latent risk factors

**DOI:** 10.1186/1471-2482-12-10

**Published:** 2012-06-22

**Authors:** Martie van Beuzekom, Fredrik Boer, Simone Akerboom, Patrick Hudson

**Affiliations:** 1 OR Centre, Leiden University Medical Centre, 9600,, 2300, RC Leiden, the Netherlands; 2 Department of Anaesthesiology, Leiden University Medical Centre, 9600,, 2300, RC Leiden, the Netherlands; 3 Department of Psychology, Leiden University, 95555, Leiden, the Netherlands; 4 Department of Safety Science, Delft University, of Technology, Jaffalaan 5, 2628BX, Delft, the Netherlands

## Abstract

**Background:**

Patient safety is one of the greatest challenges in healthcare. In the operating room errors are frequent and often consequential. This article describes an approach to a successful implementation of a patient safety program in the operating room, focussing on latent risk factors that influence patient safety. We performed an intervention to improve these latent risk factors (LRFs) and increase awareness of patient safety issues amongst OR staff.

**Methods:**

Latent risk factors were studied using a validated questionnaire applied to the OR staff before and after an intervention. A pre-test/post-test control group design with repeated measures was used to evaluate the effects of the interventions. The staff from one operating room of an university hospital acted as the intervention group. Controls consisted of the staff of the operating room in another university hospital. The outcomes were the changes in LRF scores, perceived incident rate, and changes in incident reports between pre- and post-intervention.

**Results:**

Based on pre-test scores and participants’ key concerns about organizational factors affecting patient safety in their department the intervention focused on the following LRFs: Material Resources, Training and Staffing Recourses. After the intervention, the intervention operating room - compared to the control operating room - reported significantly fewer problems on Material Resources and Staffing Resources and a significantly lower score on perceived incident rate. The contribution of technical factors to incident causation decreased significantly in the intervention group after the intervention.

**Conclusion:**

The change of state of latent risk factors can be measured using a patient safety questionnaire aimed at these factors. The change of the relevant risk factors (Material and Staffing resources) concurred with a decrease in perceived and reported incident rates in the relevant categories. We conclude that interventions aimed at unfavourable latent risk factors detected by a questionnaire focussed at these factors may contribute to the improvement of patient safety in the OR.

## Background

Patient safety is one of the greatest imperatives in healthcare today [1]. However, there are many obstacles that must be overcome to make the healthcare system truly safe. This article describes one approach to successful implementation of a patient safety program at the systemic level. A specific strategy for operationalizing a safety program is provided. Through this strategy it is possible to identify and address safety concerns proactively, to develop specific tools and resources that can be used to support an environment of safety and create mechanisms to modify the program in response to patient and staff needs as well as changing priorities. In the contrast between events that are often minor, but salient, and the major, but latent or hidden, systemic weaknesses, most attention has been devoted to the obvious problems. Success here, with individual protocols and techniques, tackles the patient safety problem one issue at a time. This article attempts to attack the deeper-seated underlying problems that, when accurately identified, allow for remedial actions that can impact whole classes of issues simultaneously.

It is increasingly accepted that adverse outcomes are often due to system failures, whereby deficiencies at many different levels create the context in which human error can have a negative impact [2-4]. Studies also have shown that organizational factors contributing to error and to safety can be grouped into a limited number of general failure classes or Latent Risk Factors (LRFs), including such error-producing conditions such as poor design, maintenance failures, unworkable procedures, shortfalls in training, less than adequate tools and equipment and inadequate staffing [5]. For example, nurse understaffing has been ranked by both the public and physicians as one of the greatest threats to patient safety in US hospitals [6]. The identification of LRFs, that may impact the expected course of care and often compromise patient safety, can support a better understanding of the operating room as a system and the identification of system components that influence patient safety [7]. A proactive systems approach to surgical safety suggests that it is necessary to study all aspects of the system that comprises a surgical operation, ranging from such issues as equipment design and use, to communication and team coordination [8,9]. Safety experts argue that proactively reducing such latent risk factors, that increase the risk of error by many individuals, will result in delivering safer care more quickly than taking measures directed, often reactively, at specific individuals [2]. Consistent with the objective of minimal patient harm, safety management in health care should be proactive rather than reactive; that is, broad risks should be anticipated and reduced before patients are harmed rather than waiting to identify specific problems and then attacking them. The question is, how can you identify such risks *before* an incident, rather than waiting for an adverse event or hoping for a report that can uncover a problem?

A proactive error management system, designed to measure and reduce the adverse impact of LRFs within an organization, may provide the answer. Proactive systems work in part by asking people to judge how frequently each of a number of factors such as staffing, supervision, procedures and communication impacts adversely on specific aspects of their work. This type of proactive approach allows the identification of LRFs before they give rise to errors that can compromise patient safety. Such a system may serve not only to reduce error, but also to foster a culture that, by moving away from blaming the individual, encourages reporting, creating a virtuous circle [10]. In the operating room (OR) errors are frequent and often consequential. In reported studies on the incidence of adverse events in hospitals, the largest number occurs in the OR. The proportion of adverse events in the operating room appears to be remarkably stable, comprising approximately 50 % of all adverse events within a hospital [1,11-13]. This suggests that the OR is a domain in which improved safety is an urgent and significant challenge. A critical first step in an improvement process involves systematically addressing those factors contributing to adverse events in the OR. Increased awareness of patient safety issues and the resources that are available to both health care practitioners and consumers can help staff ward off patient safety problems before they occur [14].

### Aim of the study

This study is prospective and is concerned with the question whether an intervention, based on a safety program, leads to improvement on latent risk factors and an increase in incident reporting. It was anticipated that concretely addressing LRFs, rather than just a general awareness campaign, will contribute to the prevention of future errors and consequently to improved patient outcomes. This article describes the results of the intervention and gives suggestions for quality improvement initiatives.

## Methods

### Setting

A pre-test/post-test control group design was used to evaluate the effects of the interventions. The staff from one university hospital operating room acted as the intervention group (I-OR). The control group which received no interventions consisted of the staff of the OR in another university hospital (C-OR). The organizations were located in the Netherlands. At baseline and again at follow-up after 1.5 year all staff (including trainees) and operating room nurses/technicians who had been in their job three months or more were approached and invited to fill out the survey. The study was approved by the Research Ethics Board of the Leiden University Medical Centre (the Netherlands).

### LOTS-study

The hypothesis that correcting LRFs, concentrating on systemic rather than individual issues, will result in safer care became the cornerstone of the Leiden Operating Theatre Safety (LOTS) project. This project aims to identify system failures in the OR irrespective of the errors and incidents directly, and to develop and evaluate interventions to reduce those failures, leading to a reduction in errors in the long term.

To assess the OR’s resistance to error a comprehensive survey instrument was developed measuring the presence of systemic failures that lie dormant in the working environment of the operating room and intensive care unit - the Leiden Operating Theatre and Intensive Care Safety (LOTICS) scale. It can be used in a pre-test, intervention, post-test design to evaluate the effectiveness of changes brought about in the hospital or a specific unit [15].

### Survey instrument

#### Latent risk factors

The approach taken to assess the state of the individual LRFs is analogous to a health check, assessing a limited number of well-chosen diagnostic vital signs. Items, presented as statements, can be indicators of either potential problems or good practice. Possessing the former or lacking the latter can both be treated as indications that there are latent failures present in a particular LRF. Failure to find indications of problems and possession of the factors that are evidence of good practice can both be treated as indications that there are no latent failures present in a particular LRF.

Latent risk factors were measured with the LOTICS-scale (Additional file 1: appendix 1) [15]. The LOTICS has been validated with respect to factor structure and reliability of the scales, as well as its content and discriminative validity, and measures 11 LRFs with a total of 51 indicator questions: Training (6 items, α = .77; e.g. “In my department, staff are well trained in the use of new equipment”), Staffing Resources (6 items, α = .81; e.g. “In my department, there are enough experienced staff”), Planning & Coordination (3 items α = .75; e.g. “In my department, only short-term plans are made”), Communication (6 items, α = .84; e.g. “Information to perform procedure is available at the time when it is needed“), Material Resources (5 items, α = .75; e.g. “In my department, material/equipment is of insufficient quality”), Maintenance (4 items, α = .81; e.g. “Maintenance inspections are carried out on time”), Design (4 items, α = .78; e.g. “Controls or displays are hard to read”), Quality of Procedures (6 items, α = .79; e.g. “In my department, procedures, rules, and guidelines are often not feasible in practice”), Teamwork (4 items, α = .74; e.g. “Members of my team work well together during the operation ”), Team Instruction (3 items, α = .80; e.g. “Team members receive sufficient instructions during the operation”), and Situational Awareness (4 items, α = .77; e.g. “There is sufficient information exchange during the surgery”). Respondents indicate their agreement on a 4-point rating scale (1 = *strongly disagree*, 4 = *strongly agree*). The same scalar structure was presented throughout the questionnaire, and then adjusted post-hoc. For all LFRs, negatively formulated items were recoded so that a higher score always indicates more favorable perceptions about organizational and environmental conditions of work.

#### Perceived incident rate

In this study, incidents are defined as all safety-related events including accidents (with negative outcomes such as damage and injury), near misses (where an accident could have happened had there been no timely and effective recovery) and errors (no harm events). We asked respondents to report how often errors, near-misses and accidents occurred in their departments. The three items were scored on a six-point scale ranging from 1 (never) to 6 (very frequently), with a higher score indicating a greater perception of incidents.

The questionnaire has an additional demographic section where respondents fill in their department or ward, job tenure on current ward (1 = <1 year, 2 = 1-5 years, 3 = 6–10 years, and 4= > 10 years), age and gender.

Finally, participants were asked about the organizational and environmental conditions that affect patient safety in their department and the possible remediable action alternatives for addressing them.

### Incident reporting

Incident data were collected and then systematically analysed using the Prevention and Recovery Information System for Monitoring and Analysis (PRISMA) - Medical method over a 12-month period before and after the intervention [16]. The PRISMA method is based on the so-called system approach to the problem of human error and therefore concentrates on the conditions under which individuals work. It was originally developed in and for the steel industry and has been applied successfully in the medical domain [16,17]. Key components are an in-depth incident analysis to detect causal factors, and the Eindhoven Classification Model to classify the root causes found into technical, organizational, human, and patient related factors.

### Intervention

A multidisciplinary safety committee (surgeons, anesthetists, operating room and recovery nurses) was created to improve incident reporting and to develop a number of measures aimed at the LRFs which need improvement.

The level of reporting of incidents was considered to be an important factor of the safety program. To improve the quality and completeness of reporting incidents and to achieve a general raising of awareness of patient safety problems we developed and implemented a voluntary electronic/web based reporting system and provided feedback to demonstrate the value of reporting by showing its effects on organizational culture and patient safety. Feedback was always provided at team level. In case of serious incidents there was also feedback at the individual level. All reports were reviewed by safety committee members, and selected reports were discussed during the monthly meetings. When required, reports were further analyzed by individual committee members according to their expertise.

The results of the pre-test formed the basis for the choice of interventions. Based on these results (see Table 1) the following LRFs were considered best targets for intervention: Communication, Material Resources, Training, Planning, and Staffing Resources. Compared to the other LRFs respondents in I-OR scored less favorably on these LRFs. On three of these LRFs, i.e. Communication, Planning and Training, the I-OR scored even lower than the C-OR (see Table 1).

**Table 1:** Mean LOTICS scores and perceived incident rate at pre-test compared for the I-OR and the C-OR (t-tests)

**LRFs of LOTIC-scale**	**I- OR N = 111 C-OR N =82**	**Mean**	***SD***	**t**	**df**	***P***	**95 %**	**CI**
Communication	I-OR	2.43	.43	-3.00	186	**.003**	-.055	.12
	C-OR	2.61	.39					
Design	I-OR	2.95	.32	.07	182	.942	-.017	.14
	C-OR	2.94	.38					
Maintenance	I-OR	2.78	.53	-1.88	174	*.061*	-.16	.06
	C-OR	2.92	.40					
Material Resources	I-OR	2.59	.35	.33	185	.745	.09	.25
	C-OR	2.57	.39					
Planning & Coordination	I-OR	2.71	.37	-2.25	183	**.026**	-.04	.13
	C-OR	2.83	.37					
Teamwork	I-OR	2.91	.32	-.33	186	.740	.07	.19
	C-OR	2.93	.29					
Procedures	I-OR	2.72	.33	-.82	184	.415	-.04	.09
	C-OR	2.74	.33					
Situation Awareness	I-OR	2.85	.41	.96	182	.338	.12	.31
	C-OR	2.79	.44					
Team instructions	I-OR	2.84	.40	-.10	178	.924	.12	.26
	C-OR	2.84	.33					
Training	I-OR	2.67	.45	-2.36	187	**.019**	-.05	.12
	C-OR	2.82	.38					
Staffing Resources	I-OR	2.71	.46	-187	100	.793	-.02	.16
	C-OR	2.81	,30					
Perceived incident rate	I-OR	3.97	.58	-.12	178	.901	-.02	.16
	C-OR	3.98	.56					

Significant values are shown in bold.

At baseline we asked participants about their concerns on patient safety in their department. A number of issues related to the LRFs studied were named. On average, three times more issues were identified for LRFs with unfavourable scores than for LRFs with favourable scores. In I-OR most problems concerned Training, Material Resources, and Staffing Resources. Given these findings and to create as much possible involvement for the intervention we decided to focus on these three LRFs. However we realized that an intervention can have effects on other LRFs beyond the three selected, because changes do not occur in isolation. Moreover, in the literature, Material Resources, Training and Staffing Resources are mentioned as important contributors to medical errors [18,19].

*Material resources* (1) Surgical adverse events are often attributable to technique-related procedures that occur during the operation, many of which are considered preventable [20][21]. Variations in equipment in its use increase the likelihood of error [22]. People may be more willing to violate safety rules because the material does not function in the way it is supposed to do, either because of poor maintenance or because of faulty design.

*Training* (2) Lack of training and experience are also mentioned as sources of medical errors, although these causes are usually not directly documented in studies of errors and incidents. Training has, however, been shown to decrease incident rates and increase the ability to solve problems, particularly for inexperienced professionals [23-25].

*Staffing resources* (3). There is little published work examining the relationship between workload and either quality or safety of anaesthetic care [26]. Staff often forms the last layer of defence for error occurrence and understaffing or insufficient staffing is a threat to patient safety in the OR [27]. Adequate staffing is fundamental to quality care; evidence is mounting that increasing the number of registered nurses results in better patient safety [28]. Higher staffing levels are associated with lower mortality outcomes in UK hospitals [29].

We started the intervention with a training session to show which errors are made in the operating room and how they can be traced back to latent risk factors. In addition, sessions were held to introduce the new electronic reporting system. Subsequently, an exercise involving standardization of materials and equipment was performed. All OR staff then received training for all the equipment used during operations. Parallel to this, a program aimed at improving nurse retention was carried out focussing on work climate characteristics like participation in decision making, job autonomy and social support. The content of the safety program is described in Table 2.

**Table 2:** Safety program

*Awareness*	To create awareness about safety, a symposium about safety was organized. Topics were: the system approach to human error safety problems in the OR and incident reporting
*Error reporting*	A local committee of the department’s anaesthesiology and surgery was set.
	Introduction of an electronic incident reporting management system accessible to all staff and easy to use.
	Providing feedback to demonstrate that reporting leads to changes.
	Errors were discussed in the team meetings.
	Every month a newsletter was distributed with information on reported errors.
	and measures taken promoting report of near misses and errors.
*Material Resources*	Inventory of all equipment and supplies of anaesthesia and surgery.
	Standardization of equipment and supplies in anaesthesia and surgery for all equipment development of manuals with a uniform design.
*Training*	Training of all OR staff in the use of equipment.
*Staffing Resources*	Increasing participation in decision making.
	Introduction of frequently held staff meeting, at least once a month.
	Increasing job autonomy shifting for a specific task responsibility and control from supervisor to staff.
	Responsibility for safety in the working environment.
	Intervision for registered nurses.
	Personal coaches assigned to trainees.
	Social activities to promote team building.
	More trainees were trained.

### Statistical analyses

The data were analysed using the statistical software package SPSS version 16. Negatively worded items were reverse scored so that their valence matched the positively worded items. T-tests were used to assess differences at pre-test between the intervention (I-OR) and control group (C-OR). Chi square analyse was used for gender and reported incidents. As a means of assessing the effects of the interventions, analyses of covariance (ANCOVA) were carried out. In the ANCOVAs, age, gender, job tenure on current ward, and pre-test scores on LRFs and perceived incident rate were used as covariates**.**

### Participation and dropout

Baseline response rate was 59 %; 193 (I-OR 111 and C-OR 82) out of 327 questionnaires were returned. The response rate at post-test was 62 %; 205 (I-OR 108 and C-OR 97) out of 333 questionnaires were returned. Of the 111 professionals in I-OR and the 82 professionals in C-OR who filled out the questionnaire at baseline, 62 in I-OR and 40 in C-OR participated at follow-up as well. At both points of measurement there were no significant differences in demographic characteristics between respondents and non-respondents. For both I-OR and C-OR applies that there were no significant differences between subjects participating only in the first or the second measurement and those who took part in both measurements on demographic characteristics, LRF scores and perceived incident rate. There was just one exception; at the pre-test in C-OR staff who participated in both measurements reported more favorable on Team Instructions than staff who participated only in the first measurement.

Comparing I-OR with C-OR at pre-test on demographic characteristics, LRFs, and perceived error rate resulted in significant differences for years of employment and age (Table 3). Staff in the intervention group was younger, had shorter job tenure and are more often female. For this reason, age, job tenure on current ward, and gender were entered as covariates in all effect analyses for the group who took part in both measurements. At the pre-test I-OR differed from C-OR on three of the dependent variables: Communication, Planning & Coordination, and Training (Table 1). Staff in I-OR reported less favourable on each of these LRFs than staff in C-OR.

**Table 3:** Demographic characteristics of the participants in the I-OR and C-OR at pre- and post-test

**Pre-test**	**I-OR N = 110**	**C-OR N = 82**	**t**	**95 %CI lower**	**upper**	***P***
Age	35.4 (10.9)	40.8 (9.4)	-3.26 (1,187)	-7.95	-1.958	.001
Employment status (hours)	31.4 ( 9.3)	31.1 (11.7)	.205 (1,185)	-2.70	3.32	.868
Job tenure	2.7 (99)	3.2 (1.02)	-.3.32 (1,185)	-.87	-1.88	.001
Gender	11 % male 89 % female	36 % male 72 % female				<.01
Post-test	I-OR n = 108	C-OR n = 97	t	95 %CI lower	upper	*P*
Age	36.2 (11.4)	40.3 (11.3)	-2.602- (1,202)	-7.30	-1.006	.010
Employment status (hours)	30.8 (10)	31.2 (9.93)	-.278(1, 202)	-3.16	2.38	.781
Job tenure	2.8 (94)	2.8 (.93)	.146 (1,203)	-.278	.240	.884
Gender	10 % male 90 % female	23 % male 67 % female				<.05

### Effects of intervention

First, changes over time in the I-OR were analyzed by comparing the results of all staff who took part at baseline with those of all staff who took part at follow-up. The results of the t-tests, pre- and post-test mean scores on LRFs and perceived incident rate for I-OR and C-OR are shown in Table 4.

**Table 4:** Mean LOTICS scores and perceived incident rate at pre-test compared for the I-OR and C-OR at post-test (t-tests)

**LRFs of LOTIC-scale**		**I-OR**	**C- OR**	**t**	**df**	***P***	**95 % CI**	
		n = 108	n = 97					
Communication	pre	2.43	2.61					
	post	2.38	2.57	-3.42	203	**.001**	-.306	-.082
Design	pre	2.95	2.94					
	post	2.99	3.03	-.749	203	.455	-.028	.058
Maintenance	pre	2.78	2.92					
	post	2.91	2.94	-.515	193	.607	-.143	.084
Material Resources	pre	2.59	2.57					
	post	2.72	2.53	3.602	202	**.000**	.085	.290
Planning & Coordination	pre	2.71	2.83					
	post	2.78	2.83	-.911	201	.363	-.143	.052
Teamwork	pre	2.91	2.93					
	post	2.93	2.92	.160	201	.873	-.091	.107
Procedures	pre	2.72	2.74					
	post	2.69	2.70	-.392	202	.696	-.103	.69
Situation Awareness	pre	2.85	2.79					
	post	2.82	2.83	-.264	192	.792	-.143	.109
Team instructions	pre	2.84	2.84					
	post	2.84	2.81	.565	190	. 573	-.087	.157
Training	pre	2.67	2.82					
	post	2.81	2.82	-.228	203	.820	-.114	.091
Staffing Resources	pre	2.71	2.81					
	post	2.84	2.73	1.989	203	**.048**	.001	.215
Perceived incident rate	pre	3.97	3.98					
	post	3.59	3.96	-4.079	202	**.000**	-.551	-.192

Significant values are shown in bold.

The I-OR rated more favorably on Staffing Resources and Material Resources at follow-up than at baseline. For the other LRFs no statistically significant changes over time were found, except for communication. This LRF scored in the I-OR less favorably at follow-up than at baseline. Finally, the I-OR scored significantly lower on perceived incident rate at follow-up than at baseline. At follow-up the C-OR rated more favorably on Design and less favorably on Staffing Resources than at baseline.

**Figure 1:**
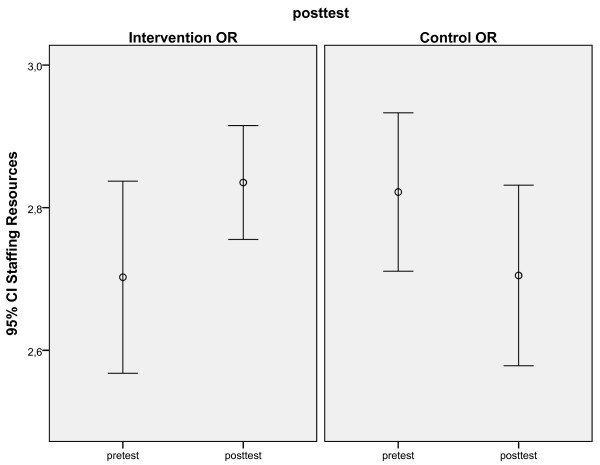
Significant differences between I-OR (1) and C-OR (2) on pre and post-test scores: Staffing resources.

**Figure 2:**
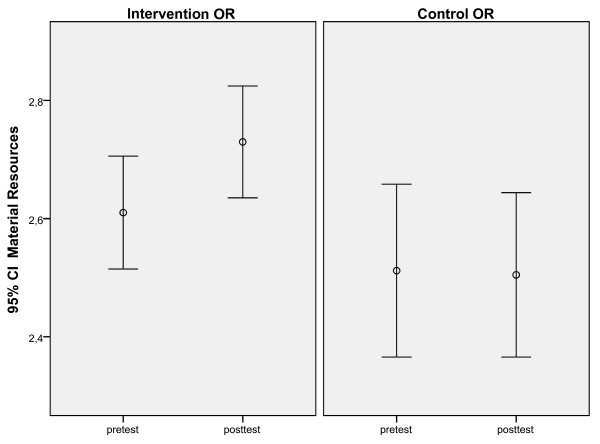
Significant differences between I-OR (1) and C-OR (2) on pre and post-test scores: Material resources.

Second, separate univariate ANCOVAs were conducted, using data from staff that participated in both measurements, to test if there had been a different development in the I-OR compared to the C-OR from pre- to post-test. The intervention had focused on three LRFs: Material Resources, Training and Staffing Resources. So, we expected at follow-up higher scores on these LRFs in I-OR, indicating fewer problems, than in C-OR. Consistent with our expectations, there was a positive effect of the intervention aimed at Staffing Resources. When pre-test scores, age, gender, and job tenure were used as covariates, a significant effect over time was found between the I-OR and the C-OR. (Table 5). Staffing resources improved in the I-OR but worsened in the C-OR from pre-test to post-test measurement (Figure 1). There was also a positive effect of the intervention aimed at Material Resources. When pre-test scores, age, gender, and job tenure, were used as covariates, a significant difference was found on Material Resources to the advantage of the I-OR (Figure 2). The intervention aimed at Training was not significant. When pre-test scores, age, gender, and job tenure, were used as covariates, there was no significant difference between the I-OR and the C-OR on Training over time.

**Table 5:** Comparison of I-OR and C-OR by separate univariate ANCOVAs (repeated measures) with pre-test scores, age, gender and job tenure as covariates

**LRFs of LOTIC-scale**		**I-OR n = 62**	**C- OR n = 40**	**F ratio**	***P***
Communication	pre	2.48	2.54	3.07	.083
	post	2.35	2.54		
Design	pre	2.94	2.96	0.26	.872
	post	2.99	2.95		
Maintenance	pre	2.81	2.98	2.43	.122
	post	2.94	2.94		
Material Resources	pre	2.60	2.51	8.38	**.005**
	post	2.73	2.50		
Planning & Coordination	pre	2.75	2.78	2.14	.147
	post	2.83	2.73		
Teamwork	pre	2.93	2.86	.167	.684
	post	2.95	2.86		
Procedures	pre	2.68	2.72	0.41	.525
	post	2.66	2.70		
Situation Awareness	pre	2.84	2.71	1.90	.171
	post	2.78	2.77		
Team Instruction	pre	2.87	2.75	0.73	.788
	post	2.84	2.74		
Training	pre	2.72	2.77	1.61	.207
	post	2.80	2.74		
Staffing Resources	pre	2.70	2.72	10.3	**.002**
	post	2.81	2.58		
Perceived Incident rate	pre	3.93	3.96	5.45	**.02**
	post	3.53	3.88		

Significant values are shown in bold.

**Figure 3:**
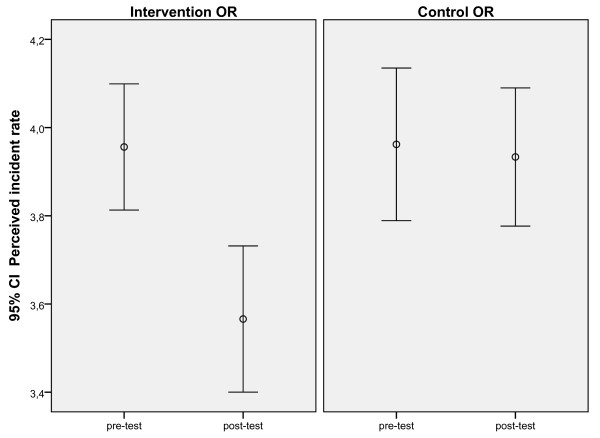
Significant differences between I-OR (1) and C-OR (2) on pre and post-test on perceived incident rate.

We also expected in I-OR a decrease in perceived incident rate. The results indeed support our hypothesis showing a significant difference between the I-OR and the C-OR in perceived incident rate over time when pre-test scores, age, gender, and job tenure were used as covariates. There was a decrease in perceived incident rate in the I-OR (Figure 3) while perceived incident rate did not change in the C-OR.

The number of reported incidents multiplied with a factor 2.4 between the pre/post intervention period. In the year before the intervention there were 250 reported errors. Of these errors 80.8 % were classified as human, 8.8 % as technical, 9.6 % as organizational, and 0.8 % as patient related. In the year after the intervention the number of reported errors increased to 629 of which were 83.9 % human, 3.7 % technical, 10.8 % organizational, and 0.95 % patient related. The increase in reported incidents was mainly due to an increase in the number of reported near misses and errors. The decrease in the contribution of technical causes, referring to physical items such as equipment, materials, instrumentation, installations, labels and forms, from 8.8 % to 3.7 % was significant (*p* = .001).

## Discussion

The study shows that our intervention aimed at Material resources, Training and Staffing resources resulted in demonstrable changes of scores on two of the relevant LOTICS scales. This type of intervention can provide direct benefits to the staff of an OR, because the changes on the working environment were both visible and resulted in improvement in task performance and are therefore likely to be accepted.

The philosophy underlying the development of the LOTICS scale is that interventions should address broad categories of error types (the underlying pathology) rather than individual symptoms. Given this approach, the intervention aimed at improving material resources was based on the concept of standardization. Standardization is a concept well understood by other safety critical industries that value the benefit of lightening the mental burden on staff and users to allow them to concentrate better on the job at hand [30]. In aviation the standardization and disciplined use of procedures, termed SOPs (Standard Operating Procedures) is widely argued to be the most critical factor distinguishing between good and poor outcomes in aviation incidents [31] and could be adapted to the OR to develop protocols that minimize the influence of competing tasks and high workload. Standardization of material and equipment further results in the reduction of costs of operation, in maintenance, repair, storage, and simplified issue procedures. As part of the process in I-OR to standardize and streamline instrumentation and equipment, including locations, old and/or less user-friendly apparatus was replaced, missing items were purchased and manuals with a uniform design were developed. This improvement (at a general level) should and did affect responses to specific test items referring to, amongst others, the availability of equipment, their quality, timely repair and replacement. Moreover, after the intervention PRISMA identified technical factors to be significantly less important as causes of incidents.

Understaffing is one of the greatest threats to patient safety. Staff are often the last layer of defence for any error occurrence and particularly the proportion of professional nursing staff has an effect on patient safety [25,32,33]. At the time of the pre-test there were shortages in OR personnel in 14 out of the 60 (23 %) Dutch hospitals investigated [33]. One of the reasons for understaffing in the Netherlands is that working in healthcare is found to be less appealing [34,35]. To limit turnover and to attract new personnel we need to enhance the attractiveness of the profession. To investigate how this can be achieved we designed and evaluated a number of intervention programs. These programs focused on the enhancement of well-studies work climate characteristics: participation in decision making, job autonomy and social support. Employee perceptions of these characteristics have been linked to various stressors, and a number of individual and organizational outcome variables [36]. In addition to the focus on work climate characteristics more training opportunities were created so that more trainees could be qualified. As expected the interventions turned out to result in higher scores in I-OR compared to C-OR on aspects like the amount of staff to provide good care and the amount of experienced staff.

Staff turnover rate in I-OR decreased from 9.4 % in the year before the intervention to 5.1 % in the year after the intervention. Although we realize that turnover is determined by many factors, including labor market, it is likely that some of this decline can be attributed to the interventions.

Change can be a complex and drawn-out process that depends on a variety of contextual factors. The OR is a highly compartmentalized department structure which brings together members from multiple disciplines whose training and professional goals vary. Lack of communication between operating room personnel is common [37]. Most surgical errors are not attributable to an individual but involve multiple personnel and steps; approximately 43 % of errors are due to poor communication [20]. During the intervention in the OR we actually saw an increase in reported problems with communication. When communication problems do occur, they are found most often between different professional members of a team, such as between anaesthesiologist and surgeon or between nurses and doctors [38].The staff of the I-OR indicated that they needed more information to do their tasks. A tentative explanation for this result could be that having created heightened awareness about safety issues, the staff was more alert to the communication problems they experienced.

The importance of incident reporting is widely recognized [10,39]. Unfortunately, reporting is grossly incomplete. After the intervention, incident reporting rates in I-OR increased significantly compared with pre intervention rates. We realize that it is difficult to deduce from this result whether the 2.4x change in error reporting reflects a change in report behaviors with actual rates remaining constant or whether the 2.4x change in error reports reflects an increase in error rates despite the intervention. Various studies, however, showed that as an institution improves in the care it delivers and its safety culture more problems may be reported since open reporting is a tenet of safe practice [40]. Increased incident reporting rates may not be indicative of an unsafe organization, but may reflect a shift in organizational culture [41]. In this context it is important to note that the total number of reported incidents more than doubled while the contribution of technical factors to incident causation remained constant.

The propensity to report is probably further strengthened in our study by the implementation of the electronic report system. Various studies showed that an accessible and easy to use reporting system [42], the understanding that the reports will be handled in a non-punitive manner [43], and the notion that the reports are taken seriously and will lead to enhanced learning and systematic changes which will prevent it from recurring [44], positively affects the willingness to report incidents. The empirical findings in this and other studies, taken as a whole, suggest that our result, an increase in incident reporting in I-OR, reflects a change in report behaviors rather than an increase in incident rates.

We believe that this work can contribute to patient safety initiatives and research in two ways: (1) our experience provides detailed insight in the latent risk factors, (2) our findings suggest that the methodology used in the study shows promise as a method for evaluating changes in the quality and safety of care in the operating rooms. Changing culture is a new watchword in patient safety [45]. The willingness of staff to speak up about a patient-safety concern is an important part of safety in the operating room [46]. Therefore there needs to be a culture of openness [47]. We think a first step is this approach is to build a strong foundation of safety awareness among your staff and this may best be done by implementing concrete and visible improvements. We think staff perceptions of safety are a high priority issue within the OR, which will eventually motivate staff to take greater ownership of and responsibility for patient safety.

### Limitation

In the present study the intervention addressing training did not result in a significant improvement. This may have been due to a failure to address the problem at a deeper level, that is, the deficiencies in the business process behind the detected indicators. It is conceivable that the intervention attacked the problem at a ‘symptom curing’ level the training of the use of new equipment. As a result, this intervention may not have remedied problems at a systemic level, as revealed by the responses to test items referring to various other aspects of the training procedure.

Safety questionnaires are increasingly used in healthcare for assessment of safety issues, but they differ in the scope and extent. Sexton and co-workers developed a safety attitudes questionnaire that was validated over a wide range of clinical areas (ICU, OR, inpatient settings and ambulatory clinics) and 3 countries and administered to a large study group [48]. The factors identified by their questionnaire were teamwork climate, safety climate, perception of management, job satisfaction, working conditions and stress recognition. They claim that the results could be used to benchmark organizations and to measure effectiveness of interventions. Similar safety questionnaires have been used by others to access teamwork and safety climate in hospitals and nursing units [49,50].

Compared to their study our study was limited to a smaller group of disciplines and settings. Furthermore our questionnaire was more limited in scope and more directed to a limited set of factors that we connected to latent risk factors (LRFs), as identified in incident analysis. But a major difference is that those LRFs assessed enabled a much more concrete identification of measures for intervention, as compared with abstract factors like the perception of management, job satisfaction and safety climate, while still providing a way of assessing pre- and post-intervention values. There is still much work required before we are able to understand the full value of using climate questionnaires in health care, as Pronovost and Sexton have [51] have recently pointed out.

## Conclusion

The change of state of LRFs can be measured using a patient safety questionnaire aimed at these factors. The change of the relevant risk factors (material and staffing resources) concurred with a decrease in perceived and reported error rates in the relevant categories. We conclude that interventions aimed at unfavourable latent risk factors detected by a questionnaire focussed at these factors may contribute to the improvement of patient safety in the OR.

## Abbreviations

ANCOVA, Analyse of covariance; C, Control; ICU, Intensive care unit; I, Intervention; LRFs, Latent risk factors; LOTS, Leiden operating theatre safety; LOTICscale, Leiden operating theatre and intensive care safety scale; OR, Operating room; PRISMA, Prevention and recovery information system for monitoring and analysis; SOP, Standard Operating Procedure.

## Competing interests

The authors declare that they have no competing interests

## Author’s contribution

MvB, FB, SA were involved with study development, co-ordination and data collection and writing the article. MvB, SA were involved with data analysis. PH has been involved in drafting the manuscript, and revising it. All authors read and approved the final manuscript.

## Pre-publication history

The pre-publication history for this paper can be accessed here:

http://www.biomedcentral.com/1471-2482/12/10/prepub

## Supplementary Material

Additional file 1 **Additional file 1: Appendix 1.**LOTICS scale . Click here for file
