# Supplementary material for: Patient safety in the operating room: an intervention study on latent risk factors
Source: BMC Surg. 2012 Jun 22;12:10. doi: 10.1186/1471-2482-12-10 (PMC3407486; doi:10.1186/1471-2482-12-10)
Supplement: Additional file 1 — Appendix 1.LOTICS scale . [file 1471-2482-12-10-S1.doc]

Appendix 1: LOTICS scale

| Communication | Information about changes in OR program / planned procedure timely provided  Information about changes in OR program / planned procedure are communicated through  the right channels  Adequate communication about patients with other disciplines  Information to perform procedure available at the time when it is needed  Adequate communication about patients between teams  Information to perform procedure not properly communicated |
| --- | --- |
| Design | Equipment operation is difficult  Controls or displays are hard to read  Controls of displays are unclear and / or lacking  Too much information on controls or display |
| Maintenance | Maintenance carried out on a regular basis  Maintenance inspection performed timely  OR / ICU equipment badly maintained  Maintenance schedule is lagging |
| Material Resources | Following new technologies when procuring new equipment  Availability of materials & equipment at the time it is needed  Insufficient quality of materials & equipment  Worn-out or faulty equipment replaced in a timely way  Equipment frequently repaired  Instruments often incomplete |
| Planning & Coordination | Organizational changes not adequately supported within the department  Lack of advance planning within the department  Sufficiency of planning |
| Teamwork | I really feel I am a part of my team  Team’s ability to deal with unexpected events  Members of my team work together as a well coordinated team  Clear view of who is doing what and when |
| Procedures | Accessibility of procedures / regulations / rules  Violations of procedures / regulations / rules  Procedures / regulations / rules frequently not clear  Procedures / regulations / rules frequently not applicable in practice  Procedures / regulations / rules applied correctly  Procedures taken a bit less seriously to do a better job |
| Situation Awareness | Team members alert each other to problems  Members of my team know what one another is doing  Members of my team monitor each others performance  Adequate exchange of information during the operation / shift |
| Team Instructions | Team members debriefed on what they can expect during operation / shift  Team members sufficiently instructed during operation / shift  I have confidence in my other team members |
| Training | Adequate coaching of new personnel  Keeping employees informed about new medical / technological developments  Training employees in the operation of new equipment  Adequate supervision of trainees in their practical period  Co-workers on my department have the necessary qualifications  In OR combination of staff junior / junior are avoided // on the ICU an adequate mix of  seniority is applied |
| Staffing Resources | Enough staff to provide good care  Enough physicians to provide good care  Enough support staff to provide good care  Enough experienced staff available  Bringing in replacement staff during absence leave  Enough experienced physicians available |
